# Characterization, *in vitro* antioxidant activity and stability of cattle bone collagen peptides‑selenium chelate

**DOI:** 10.1016/j.fochx.2024.101789

**Published:** 2024-08-30

**Authors:** Jian-Ming Li, Wen-Jun Wang, Hui Chen, Su-Yun Lin, Xin-Yi Mao, Jun-Min Yu, Ling-Li Chen

**Affiliations:** College of Food Science and Engineering, Jiangxi Agricultural University, Nanchang 330045, China

**Keywords:** Selenium-chelating peptides, Selenium binding capacity, Structural characteristics, Antioxidant property, Stability

## Abstract

In this study, cattle bone collagen peptides–selenium chelate (CCP-Se) was prepared and its structure, oxidation resistance and stability were characterized. The selenium binding capacity was 33.65 ± 0.13 mg/g by optimized preparation conditions. Structural analysis showed that selenium ions bound mainly to the amino nitrogen, carboxyl oxygen and hydroxyl oxygen atoms of the cattle bone collagen peptide (CCP). The microstructure and particle size analyses showed that the particle size of CCP-Se was increased and formed a regular and compact crystal structure compared with CCP. Additionally, CCP-Se exhibited excellent antioxidant activity. Stability analysis showed that CCP-Se was stable in thermal processing, simulated *in vitro* digestion and acid/alkali tolerance tests. The intestinal selenium permeability of CCP-Se was significantly better than sodium selenite (*p* < 0.05). This study provides reference for the high-value application of cattle bone and suggests the potential of CCP-Se as a new effective selenium supplement.

## Highlights

• The CCP-Se was prepared and selenium binding capacity was 33.65 mg/g.
• CCP-Se had a compact structure with good thermal and pH stability.
• CCP-Se showed excellent antioxidant activity.
• CCP-Se exhibited better intestinal permeability than sodium selenite.

## 1 Introduction

Selenium (Se) is an essential trace element for human growth and metabolism. Selenium protect cellular antioxidant defense and possesses bioeffects such as anti-inflammation, anti-cancer, anti-diabetic, and cardiovascular and liver protective effects arising from Se-enhanced cellular antioxidant activity (Qi, Duan, and Ng, 2024). Low Se or Se deficiency status may cause various disorders, such as impaired fertility and immunity, cardiovascular and cerebrovascular disease, and postnatal maladjustment syndrome (Li, Chen, et al., 2018). It is estimated that one billion people are directly affected by Se deficiency on a global scale due to low dietary Se intake (Jia et al., 2024; Li et al., 2021). It is urgently needed to develop scientific and reasonable Se supplements for these Se-deficient people. In addition, excessive intake of Se can be physiologically toxic to the body, causing irreversible damage, chronic Se toxicity is observed in humans when intake of Se above levels of 400 μg/day (Li et al., 2021; Qi, Duan, and Ng, 2024). Therefore, a reasonable supplementation of Se plays an important role in maintaining the healthy status of the body.

The sources of Se supplements mainly include two forms of inorganic (*e.g.*, Se(IV) and Se(VI)) and organic selenium (*e.g.*, selenocysteine (SeCys), selenomethionine (SeMet)) (Zhang et al., 2020). Considering the low bioavailability and high cytotoxicity of inorganic Se, it is usually converted to organic Se for utilization by biochemical transformation methods (Chen et al., 2023). Organic Se can be obtained from some plants, animals and microorganisms (*e.g.*, *Cardamine violifolia* (Wang et al., 2023), selenium-enriched eggs (Chen et al., 2023), selenium-enriched yeast (Chen et al., 2023)). Compared with inorganic Se, organic Se exhibits higher bioactivity and a wider range of safe concentrations, therefore, the development of organic Se supplements has attracted much attention (Qi, Duan, and Ng, 2024). As the biological transformation time is long and the transformation efficiency is unstable, in recent years, many studies have found that the synthesized peptides‑selenium chelate can not only improve the bioavailability and stability of selenium, but also enhance the antioxidant and other biological activities, which is a promising source of organic Se supplements (Qin et al., 2020; Zeng et al., 2023). For example, Jia et al. (2024) prepared chelated abalone visceral peptides chelate (AVP-Se) and demonstrated that the formation of AVP-Se enhanced its digestion stability and thermal stability. Ye et al. (2019) prepared and confirmed that soybean protein isolate peptides‑selenium chelate (SPIP-Se) not only exhibited stronger hydroxyl radical scavenging and reducing ability than SPIP *in vitro*, but also can repair H_2_O_2_-induced oxidative damage in Caco-2 cells by enhancing antioxidant enzyme activity *in vivo*. This made SPIP-Se to be considered as a new-type of selenium supplement.

As one of the most commonly processed by-products of beef, cattle bones are often discarded in industrial processing due to its low utilization value, which not only causes a waste of resources, but also brings certain environmental pollution pressure (Lv et al., 2023). Studies have confirmed that cattle bone is rich in collagen, about 57.5 ± 8.7 mg/g (Zhang, Zhao, et al., 2021). Cattle bone collagen peptides (CCP) have the biological activities of anti-osteoporosis, promoting the proliferation of osteoblasts and inhibiting bone loss, which is important for the improvement of human health (Wang et al., 2020; Zhang, Zhao, et al., 2021), this suggests that cattle bone may be an important source of natural active ingredients. Therefore, the high-value utilization of cattle bone resources is of great significance for improving economic efficiency, environmental protection and human health.

Recent studies have shown that CCP combined with calcium can facilitate calcium absorption and improve the bioactivity of calcium ions (Zhang, Zhao, et al., 2021; Zhang et al., 2023a). For example, Qi et al. (2023) confirmed that different doses of Glu-Tyr-Gly-Ca significantly increased the calcium transport activity of Caco-2 cell monolayers and the effect was better than that of inorganic CaCl_2_. This suggested that Glu-Tyr-Gly-Ca promoted intestinal calcium absorption. It is interesting that phosphorylation modification of CCP enhanced its effect on mineralization of MC3T3-E1 cells by improving calcium-binding capacity (Qi, Wang, et al., 2024). In addition, the chelation of CCP and Mg(II) significantly increased its free radical scavenging and intracellular antioxidant enzyme activity (Zhang et al., 2023b). CCP shows many advantages in chelating with minerals, however, there are few reports on the chelation of CCP with Se. Therefore, in this study, CCP and sodium selenite were selected as raw materials to prepare cattle bone collagen peptides‑selenium chelate (CCP-Se), and its structural characteristics, stability and antioxidant activity were investigated. The peptides‑selenium chelate may enhance the bioactivity of CCP and increase the utilization rate of Se. This study would be beneficial to increase the added value of cattle bone and develop the CCP-Se as a potential new selenium supplement.

## 2 Materials and methods

### 2.1 Materials

CCP (≤1000 Da) was purchased from Yolanda Biotechnology Co, Ltd. (Shanxi, China). Sodium selenite (Na_2_SeO_3_) was purchased from Sigma-Aldrich (St. Louis, Mo., USA). Dialysis bag (3500 Da) was purchased from Beijing Solarbio Technology Co., Ltd. Unless otherwise indicated, all the other chemical reagents were analytical grade.

### 2.2 Preparation of CCP-S**e**

The preparation method of CCP-Se was based on previous reports (Ye et al., 2019; Jia et al., 2024), with a slight modification. The four single-factor variables (mass ratio of CCP/Se, pH, temperature, incubation time) affecting the selenium binding capacity were optimized. Then, based on the single-factor experiment results, three key variables were selected and then optimized using Box-Behnken Design (BBD).

The optimum chelation conditions were briefly as follows: the mass ratio of CCP/Se 1:1, pH 10.0, temperature 65 °C and incubation time 60 min. When the chelation reaction was completed, the mixture was cooled to room temperature and centrifuged at 4000*g* for 10 min, the supernatant was precipitated by the addition of 5 times the *v*olume of 95 % ethanol. After standing for 12 h, the mixture was centrifuged at 4000*g* for 10 min. The precipitate was performed multiple washes with excess anhydrous ethanol before drying in a low-temperature natural drying oven to volatilize the remaining ethanol. The obtained CCP-Se was subsequently freeze-dried for further in*v*estigation.

### 2.3 Selenium binding capacity assay

The selenium binding capacity was determined with reference to the method of Jia et al. (2024), with a slight modifications. Briefly, 0.1 g of CCP-Se was digested with a mixed acid (perchloric acid: nitric acid, 1:4, *v/v*), then the mixtures were heated using an electric heating furnace until clarified. The digestive solution was cooled to room temperature and diluted to 50 ml with Milli-Q water after adjusting to the pH 7. Then, the above solution (4 mL) was diluted to 40 mL with Milli-Q water and adjusted to pH 2.5 with HCl (6 M). The digested CCP-Se solution, edetate disodium (EDTA-2Na) (2 mL, 5 %, *w/v*), and 3,3′-diaminobenzidine (DAB) (2 mL, 0.5 %, *w/v*) were well-mixed and incubated in the dark for 40 min. Subsequently, the reaction solution was adjusted to pH 7.0, then 10 mL of toluene was added and shaken for 2 min. The toluene layer was collected and analyzed with a spectrophotometer at 420 nm after static layering. The selenium binding capacity was calculated as follows:

(1) $$\text{Selenium binding capacity}\;\left(\mathrm{mg}/g\right)=\frac{\mathrm{CV}}{\mathrm{MN}}$$

where C is the concentration of selenium calculated from the standard curve (1–10 μg/mL); V is the volume of sample solution (mL); M is the mass of the sample (g); N is the ratio of the volume fraction sample to the total constant volume.

### 2.4 Structure characterization

#### 2.4.1 Ultraviolet-visible (UV) spectroscopic measurement

The CCP and CCP-Se were dissolved in Milli-Q water at a concentration of 1 mg/mL. The UV spectra of CCP and CCP-Se were recorded using a UV spectrophotometer (UV-5200PC, Shanghai Analytical Instruments Co., LTD, Shanghai, China) in the wavelength range of 200 to 400 nm.

#### 2.4.2 Fluorescence spectroscopic measurement

The CCP and CCP-Se were both prepared in Milli-Q water at a concentration of 1 mg/mL. The fluorescence was measured by a fluorescence spectrophotometer (970CRT, Shanghai Precision Scientific Instrument Co., LTD, Shanghai, China) with the excitation wavelength of 320 nm and the emission wavelength of 350–600 nm.

#### 2.4.3 Fourier transform infrared (FTIR) spectroscopic measurement

The mass ratio of CCP or CCP-Se/KBr 1:100 was mixed, and then the mixture was ground evenly in an agate mortar in a dry environment and pressed into transparent round flakes by an infrared tablet press. The FTIR spectra of CCP and CCP-Se were recorded in the wavenumber range of 4000 cm^−1^ to 400 cm^−1^ by a FTIR spectrometer (Nicolet IS 10, America) at a resolution of 4 cm^−1^.

#### 2.4.4 Circular dichroism (CD) measurement

The CCP and CCP-Se were both prepared in Milli-Q water at concentrations of 0.5 mg/mL. The spectra were measured by CD (MOS 450 Bio-Logic Corporation Frace) at room temperature at 190–250 nm with a path length of 0.1 cm, and repeated three times. All the results had background subtracted.

#### 2.4.5 Scanning electron microscopy (SEM) measurement

The microstructure of CCP or CCP-Se was analyzed by a scanning electron microscope (JSM 6701F, JEOL, Japan). An appropriate amount of dried powder sample of CCP or CCP-Se was evenly applied to the conductive plate, sprayed with a gold-plated film, and the voltage was applied to clear focus, capturing images at a magnification of 500, 9000 and 20,000 times.

#### 2.4.6 Particle size measurement

The CCP and CCP-Se were dissolved in Milli-Q water to configure a sample solution at a concentration of 2 mg/mL, and the particle size of the CCP and CCP-Se was determined by a particle size distribution analyzer (Zetasizer Nano ZS90, Malvern Instruments Ltd., Malvern, UK) at room temperature.

### 2.5 Thermogravimetry (TGA)

The thermal properties of CCP and CCP-Se were determined by a thermal gravimetric analyzer (Discovery TGA55, PerkinElmer, USA). Under a N_2_ atmosphere, the temperature was increased from 25 to 800 °C at a rate of 10 °C/min.

### 2.6 Determination of *in vitro* antioxidant activity

#### 2.6.1 Hydroxyl radical scavenging activity

Hydroxyl radical(•OH)scavenging ability was determined on the basis of the method of Ye et al. (2019), with slight modification. Briefly, different concentrations of sample solution, 9 M salicylic acid (ethanol solution), and 9 M FeSO_4_ solution were evenly mixed at a ratio of 1:1:1, and then one volume of 8.8 M H_2_O_2_ solution was added to start reaction, Vitamin C (Vc) was used as the positive control. The absorbance was measured at 510 nm after incubation at 37 °C for 30 min. The •OH scavenging rate was calculated as follows:

(2) $$\mathrm{OH}\;\text{scavenging rate}\;\left(\%\right)=[1-(A_{1}-A_{10}/A_{01}]\times 100$$

where A_01,_ A_1_, and A_10_ are the absorbances of the blank group, the sample solution, and the background group, respectively.

#### 2.6.2 ABTS radical scavenging activity ability

ABTS cation radical (ABTS•^+^) scavenging ability was performed according to Zeng et al. (2023), with slight modification. Briefly, 7 mM ABTS solution was mixed in an equal volume of 2.45 mM K_2_S_2_O_8_ solution and incubated in the dark for 12–16 h. The ABTS solution should be diluted with PBS (pH 7.4) before using to ensure an absorbance of 0.7 ± 0.02 at 734 nm. Then, one volume of sample solution was mixed with three volumes of the ABTS solution to react for 6 min in the dark, Vc was used as the positive control. The absorbance was measured at 734 nm. The ABTS•^+^ scavenging rate was calculated as follows:

(3) $$\text{ABTS}•{}^{+}\;\text{scavenging rate}\;\left(\%\right)=[1-(A_{2}-A_{20}/A_{02}]\times 100$$

where A_02,_ A_2_, and A_20_ are the absorbances of the blank group, the sample solution, and the background group, respectively.

#### 2.6.3 Determination of ferric reducing activity

The ferric reducing power measurement was determined with reference to the method of Wang et al. (2023), with some modifications. The different concentrations of sample solution, 0.2 mM PBS solution (pH 6.6), and potassium ferricyanide solution (1 %, *w/w*) were mixed and incubated at a ratio of 1:1:1 at 50 °C for 20 min. After cooling rapidly, one volume trichloroacetic of the acid (10 %, *w/w*) was added. Subsequently, the above mixture solution was mixed with Milli-Q water and FeCl_3_·6H_2_O (0.1 %, *w/w*) at a ratio of 1:1:0.2 to react for 10 min in the dark, Vc was used as the positive control. The absorbance value was determined at 700 nm. The reducing power activity was calculated as follows:

(4) $$\text{Ferric reducing activity}=A_{3}-A_{03}$$

where A_3_, A_03_ are the absorbances of the sample solution and the blank group, respectively.

### 2.7 Thermal and pH stability

The CCP-Se was dissolved in Milli-Q water with a concentration of 5 mg/mL, heated at 25, 40, 55, 70, and 85 °C for 2 h, respectively, then cooled to room temperature and transferred to a dialysis bag (3500 Da) to remove the free selenium, the selenium content of the sample was determined as described in 2.3. The CCP-Se was dissolved in Milli-Q water with a concentration of 5 mg/mL, adjusted to pH 3, 5, 7, 9, and 11 with NaOH (1 M) or HCl (1 M), respectively, and then incubated at 37 °C for 2 h, the selenium content was determined according to the method described above. Stability was expressed by measuring the retention rate of selenium ions and calculated as follows:

(5) $$\text{Selenium retention rate}\;\left(\%\right)=\frac{m_{1}}{m_{2}}\times 100$$

where m_1_ and m_2_ represent the mass of selenium ion inside the dialysis bag and in the solution, respectively, mg.

### 2.8 Simulation of oral gastrointestinal digestion *in vitr**o***

The preparation of the simulated digestive fluid *in vitro* was based on the method described by Ma et al. (2022), and the simulated gastrointestinal digestion test was performed according to Zeng et al. (2023), with a little modification. Briefly, the pre-heated (37 °C) sample solution was added to the artificial saliva (containing 0.075 % NaCl, 0.015 % CaCl_2_, 0.15 % KCl, and 0.42 % α-amylase, *w/v*) in a proportion of 1:1 (*v/v*), the pH of the above mixture was maintained at 6.8 and oscillated digestion was done at 37 °C for 10 min. The enzyme was deactivated at 100 °C water bath for 10 min and then cooled to room temperature. The selenium retention rate of the samples was measured after digestion in the oral cavity. The pH of the above oral digestion product was adjusted to 2.0 with HCl (1 M), then the artificial simulated gastric fluid (containing 0.32 % NaCl, 0.015 % CaCl_2_, 0.11 % KCl, and 0.035 % pepsin, *w/v*) was added in a proportion of 1:1 (*v/v*) and oscillated digestion was done at 37 °C for 2 h. The enzyme was deactivated in a 100 °C for 10 min and then cooled to room temperature to measure the selenium retention. Next, the pH was adjusted to 7.4 with NaOH (1 M), then the artificial simulated intestinal fluid (containing 0.5 % NaCl, 0.035 % CaCl_2_, 0.065 % KCl, and 0.015 % trypsin, 0.4 % bile salt, *w/v*) was mixed with the above stomach digestion sample in a proportion of 10:3 (*v/v*) and placed at 37 °C for 2 h oscillating digestion. The enzyme was deactivated in a 100 °C for 10 min and then cooled to room temperature. The digestive samples of different periods were transferred to dialysis bags for dialysis to remove free selenium (Udechukwu et al., 2018). The selenium content was determined in accordance with Section 2.7.

After adjusting the pH of the above stomach digestion sample to 7.4, the artificial simulated intestinal fluid was added in a ratio of 10:3 (*v/v*), and then the mixture was transferred into the dialysis bag to oscillate and digest in a water bath at 37 °C. The selenium content of the dialysate solution outside the dialysis bag was measured at various times (0, 30, 60, 90, and 120 min) to indicate the selenium ion content permeating the simulated intestine. The selenium dialysis rate was calculated as follows:

(6) $$\text{Selenium dialysis rate}\;\left(\%\right)=\frac{m_{3}}{m_{4}}\times 100$$

where m_3_ and m_4_ represent the mass of selenium ion outside the dialysis bag and in the solution, respectively, mg.

### 2.9 Statistical analysis

All experiments were repeated at least three times and results were expressed as mean ± standard deviation. Statistical analysis was performed using SPPS 27.0 (SPSS Inc., Chicago, IL, USA). Box-Behnken Design was analyzed using Design-Expert 13.0. One-way analysis of variance (ANOVA) followed by Duncan's new multiple range tests was applied. The significance level at *p* < 0.05 was established.

## 3 Results and discussions

### 3.1 Selenium-binding capacity analysis

In order to optimize the optimal selenium chelation conditions for CCP-Se, the effects of chelation temperature, mass ratio of CCP/Se, pH, incubation time on the selenium binding capacity of the CCP-Se were evaluated. As shown in Fig. S1A, the selenium binding capacity increased when pH was increased from 5 to 11, and the maximum value was approached to 27.37 mg/g. This was probably because the coordination of selenium ions with NH^3+^ and COOH^−^ was enhanced as the chelating pH increased, which was conducive to chelate reaction (Huang et al., 2021). However, under strong acidic and alkaline conditions, the structure of CCP was unfolded and its intermolecular interactions were weakened (Du et al., 2023), the change of the coordination conformation of the peptide chain, thus reducing the selenium binding capacity. As shown in Fig. S1B, the temperature played an important role on the reaction between the CCP and selenium. With the increase of incubation temperature, the selenium binding capacity gradually increased, and reached the maximum value of 31.55 mg/g at 70 °C. As the temperature was further increased, the selenium binding capacity decreased, this may be that peptides‑selenium chelation reaction was an endothermic spontaneous process, when the temperature was too high, resulting in the unstable decomposition of CCP-Se (Ye et al., 2019). As shown in Fig. S1C, the selenium binding capacity reached the maximum value of 32.25 mg/g when incubation time was 60 min. When the chelating time was 60–100 min, there was no significant difference, which indicated that the reaction of CCP with selenium ions was rapid and could reach the equilibrium state quickly (Zhang, Ding, and Li, 2021). As shown in Fig. S1D, the selenium binding capacity first increased and then decreased, and reached the maximum of 32.88 mg/g when the mass ratio was 1:1. The mass ratio was an important condition to affect the chelation of peptide and selenium ions, when the mass ratio was too large, the ligand would be excessive, thus resulting not only the low Se binding efficiency, but also the waste of raw materials, which was not conducive to the economic efficiency (Huang et al., 2021).

According to the results of single factor, three factors (pH, chelation temperature, mass ratio of CCP/Se) were selected for subsequent response surface analysis. A statistical design of experiments was used to random Box-Behnken design (BBD) experiments at different combinations of these parameters. The design scheme and results of the BBD central combination experiment using design-expert 13.0 were given in Table S1.

The variance of each factor was analyzed using Design Expert 13.0 to verify the correctness of the model coefficients, and a quadratic regression equation model was developed using selenium binding capacity as the response value. The applicability of the model was good with model *p* < 0.01, R^2^ pred and Adjusted-R^2^ of 0.9717 and 0.9353, respectively. Therefore, the model was high significance and statistical significance. A multiple regression was fitted to the experimental data in Table S2 and the multiple linear regression equation established was Y = 32.85–1.15 × _1_–0.564 × _2−_0.3465 × _3_ + 1.38 X_1_X_2_ + 0.1416 X_1_X_3−_0.2763X_2_X_3−_3.55 × _1_^2^ - 1.49 × _2_^2^ - 1.88 × _3_^2^. Where X_1_ was the pH, X_2_ was the chelation temperature and X_3_ was the mass ratio of CCP/Se.

Through the data processing of the regression analysis results, the response surface and contour plots were made, as shown in Fig. S2A—C. The maximum *v*alue of the response surface value appeared, and the contour line was elliptical, which indicated that there was a significant interaction relationship between the parameters. The response surface test model analysis identified the maximal selenium binding capacity of 33.07 mg/g was measured by chelating at pH 10.56, temperature 67.15 °C, mass ratio 1:1, and incubation time 60 min. In order to be suitable for experimental operation, the experimental conditions were adjusted to pH 10, temperature 65 °C, mass ratio 1:1, reaction time 60 min, according to these chelation conditions, the selenium binding capacity was 33.65 ± 0.13 mg/g, which was relatively similar to the predicted value, indicating that the model can be used in practice. The results showed that the obtained optimum conditions were as follows: the mass ratio of sodium selenite to peptide was 1:1 (*v/v*), and the pH was 10, the temperature was 65 °C, and the reaction time was 60 min. The selenium chelating ability of prepared CCP-Se can reach a maximal value of 33.65 ± 0.13 mg/g.

### 3.2 UV spectroscopic analysis

**Fig. 1:**
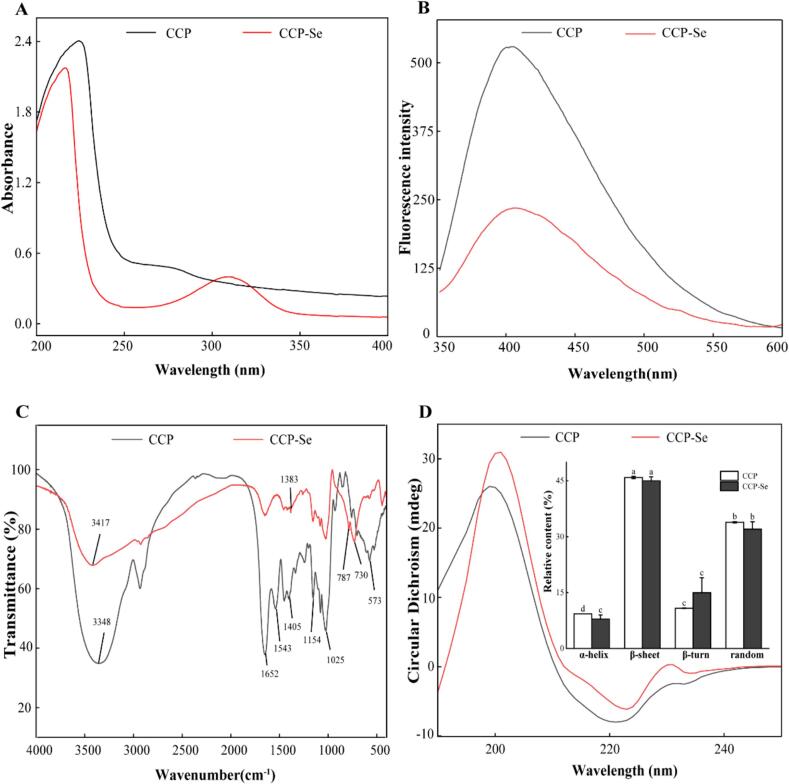
Structural characterization of the CCP and CCP-Se. (A) The UV absorption spectra; (B) The fluorescence spectra; (C) The FTIR spectra; (D) The CD spectra.

The formation of complexes of organic ligands and metal ions may lead to the shift/disappearance of pre-existing UV absorbance peaks or the emergence of new ones (Sun, Cui, Lin, et al., 2017). As shown in Fig. 1A, a maximum absorption peak was observed at 225 nm in the UV spectrum of CCP, which migrated to 217 nm after chelating with selenium. It was speculated that the carbonyl group on the peptide bond undergone an N → π* transformation after selenium was paired with the N and O bonds on the peptide chain, leading to a blue shift in the absorption wavelength (Jia et al., 2024). The CCP had a weak absorption peak at 278 nm, the absorption peak disappeared and a new absorption band was generated at 312 nm after chelating with selenium. A possible explanation was the charge migration in the aromatic amino acids as the chelation process occurred, the electron transition changed the framework structure of the protein resulting in a change in UV absorption (Qin et al., 2020).

Some studies had shown that the spatial structure with the chirality of the chromophore (C=O, -COOH) and the auxochrome (-NH_2_, -OH) of peptides would be changed after the chelation of peptides with selenium, resulting in changes in the intensity of the UV spectrum peak and shifts in the position of the absorption peak (Huang et al., 2021; Sun, Cui, Lin, et al., 2017). The carbonyl or amino group in the amide bond formed a coordination complex with selenium ions, which can explain the intensity fluctuations in this frequency band (Qi, Wang, et al., 2024). These band changes indicate that CCP-Se was a compound different from CCP.

### 3.3 Fluorescence spectroscopic analysis

Fluorescence spectroscopy is a classical method to characterize protein structural changes or protein molecular interactions. Endogenous fluorescence can be generated at specific excitation wavelengths by aromatic amino acids such as Trp, Tyr and Phe in protein molecules (Sun, Cui, Lin, et al., 2017). The fluorescence spectra of CCP and CCP-Se were shown in Fig. 1B. The fluorescence spectra of CCP-Se showed a significantly decreased in fluorescence intensity and obvious redshift. This may be due to the chelation of selenium with CCP resulting in folding and coiling of the peptide chains. It was speculated that due to the addition of selenium, the conformational change of protein causes Trp to be exposed to the molecular surface, and the polarity of the microenvironment increases, resulting in redshift and fluorescence quenching (Muhammad et al., 2022). Moreover, fluorescence quenching can also be caused by the addition of mineral ions. This phenomenon was similar to that of the chelating of egg white peptides with iron ions, which also showed the decreased fluorescence intensity (Sun, Cui, Li, et al., 2017).

### 3.4 FTIR spectroscopic analysis

The FTIR spectrum is a common method to explore secondary structure of peptides and can be used to research vibration information of various amino acid residues. The interaction between metal ions and organic ligand groups in peptides is effectively reflected by changes in the characteristic absorption peak of FTIR (Chen et al., 2014). The FTIR spectrum of CCP and CCP-Se were shown in Fig. 1C. The 3348 cm^−1^ absorption was the stretched vibration of the O—H and N—H in the CCP, and the chelation resulted in a shift to a higher frequency of 3418 cm^−1^. Due to the inductive effect or dipole field effect, the electron cloud density of N—H and O—H in CCP became stronger (Wang et al., 2018), which suggested that the oxygen and nitrogen atoms could form coordination bonds with selenium through the provision of electron pairs (Zhang, Zhao, et al., 2021). The infrared absorption of CCP in the amide I band, caused by the stretching vibration of C=O disappeared after chelation with selenium ions, which may be caused by the carboxyl group in the peptide participating in the coordination of selenium ions. The strong absorption peak at 1543 cm^−1^ was the characteristic spectrum band of the amide ІІ band, which originated principally from the N—H deformation and C—N stretches, and was displaced to a weak peak (1557 cm^−1^) after the addition of selenium (Qi et al., 2023). This also suggested that N—H participated in the chelation of selenium and formed coordination bonds with selenium. The peak changed from 1405 cm^−1^ to 1383 cm^−1^, indicating that -COO^-^ may be bound to selenium (Sun et al., 2020). In addition, the chelation site of CCP with selenium was identified as a C—Se bond by the new absorption peaks at 787 cm^−1^ and 730 cm^−1^ (Lin et al., 2021). Overall, the formation of CCP-Se resulted in a shift in the peak value, and selenium ions chelated CCP via the sites of amino nitrogen, carboxyl oxygen and hydroxyl oxygen atoms.

### 3.5 CD analysis

CD is usually used to determine the secondary structure of proteins and ligand binding of peptides (Qin et al., 2020). The CD spectra of CCP and CCP-Se were shown in Fig. 1D, the secondary structure of CCP and CCP-Se was found to have multiple conformations simultaneously based on the position and shape of the absorption peaks. A positive absorption peak near 200 nm, and a negative absorption peak between 220 and 223 nm were represented in CCP and CCP-Se. As the chelation reaction progressed, the absorption peak of CCP-Se was red-shifted. CCP has a positive peak at 199 nm and a negative at 220 nm, revealing that CCP had α-helix and β-sheet structure (Zeng et al., 2023). The secondary structures distribution assay was depicted in Fig. 1D. The proportions of β-sheet and random coil were 45.90 % and 33.90 % in CCP, respectively. The proportions of α-helix, β-sheet and random coil were slightly decreased after binding with selenium, while the proportions of β-turn structure rose by 4.15 %. Overall, there was a significant shift in the secondary structure of the peptide chain, the increase of β-turn and the reduction of random coil indicated that the structure of CCP-Se was more ordered and compact. This finding was similar to the changes in the chelating of abalone visceral peptides with selenium ions (Jia et al., 2024).

### 3.6 SEM analysis

**Fig. 2:**
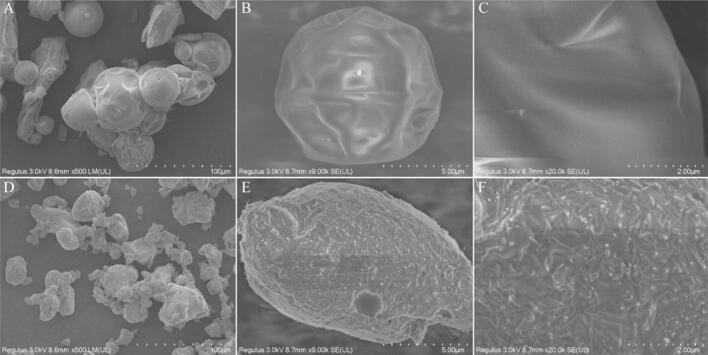
Scanning electron micrographs of the CCP (A, B, C) and CCP-Se (D, E, F) at different magnifications (A, D × 500; B, E × 9000; C, F × 20,000).

SEM is a classical approach that mainly used to evaluate the morphological characteristics of samples (Sun et al., 2021). As shown in Fig. 2, SEM was applied to observe the microstructure of CCP and CCP-Se. The surface of CCP was smooth and uneven and the structure was loose, while the crystal structure was regular and tight after chelation with selenium. The addition of metal ions was reported to affect peptide aggregation, disrupting the original structure and forming new materials, the formation of coordination bonds between the peptide and the selenium ion, resulting in the denser structure of CCP-Se (Wang et al., 2020). In addition, the particle of the CCP-Se was significantly enlarged, which was attributed to the significant effect of the addition of mineral ions on the aggregation of the peptide. Therefore, it was speculated that CCP occurred coordination binding reaction with selenium, which was mainly a folding and aggregation reaction of peptide molecules bound to selenium ions through ligand ionic bonding. This was similar to the result reported by Sun, Cui, Jin, et al. (2017).

### 3.7 Particle size analysis

**Fig. 3:**
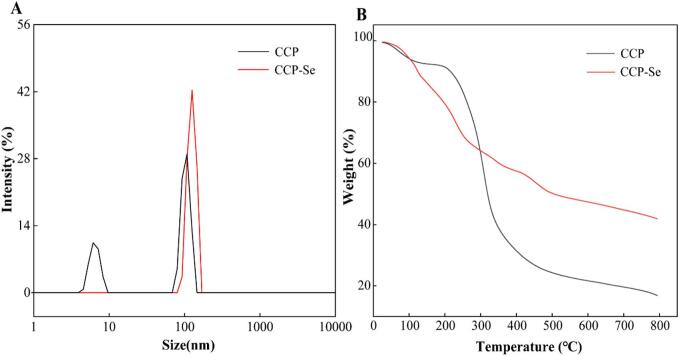
(A) Particle size distribution of CCP and CCP-Se; (B) TGA of CCP and CCP-Se.

One of the key physical parameters used to characterize metal chelating peptides is particle size (Ding et al., 2024). The average particle size distribution of CCP and CCP-Se was shown in Fig. 3A. The particle size of the CCP (382.60 ± 36.83 nm) was significantly lower than that of the CCP-Se (529.30 ± 13.46 nm). Structural folding and aggregation reactions occurring during the binding process between CCP and selenium ions may affect its particle size (Jia et al., 2024). The PDI of CCP (0.43 ± 0.035) and CCP-Se (0.62 ± 0.018) chelate were both approximately 0.5, suggesting that both of the two substances particle distributed evenly (Sun et al., 2021). This result was similar to egg white peptide-ferrous chelate (Li, He, et al., 2018). In addition, this was consistent with SEM results that the presence of selenium ions caused peptides predisposed to folding and aggregation to form CCP-Se (Jia et al., 2024).

### 3.8 Thermogravimetric analysis

The TGA curves of CCP and CCP-Se were shown in Fig. 3B. The chemical structure differences can be determined by analyzing the thermal decomposition rates of CCP and CCP-Se under different temperatures (Qu et al., 2022). There was no significant difference between CCP and CCP-Se in the initial heating stage (temperature 25–120 °C), which was mainly due to the evaporation of free water in the substance during the heating process. As the temperature continued to rise, the weight loss rates of the CCP and CCP-Se were similar at 300 °C. The chemical structure changes and the unstable polymer was thermally decomposed at this stage. When the temperature continued to rise, the sample appeared a large weight loss peak, and the weight decrease rate of CCP was more rapid than CCP-Se. At this stage, the chemical structure changed, unstable polymers undergone thermal decomposition, the non-covalent bonds including hydrogen bonding, hydrophobic interactions, and covalent bonds including C—N, C(O)-NH and C(O)-NH_2_ were decomposed (Qu et al., 2022). The mass residues of CCP and CCP-Se at 800 °C were 16.86 % and 41.89 %, respectively. Throughout the process, CCP was more affected by temperature. The reason may be that CCP forms more stable ligand bonds when chelated with selenium, which required more energy to break the chemical bond (Zhang et al., 2023a). This suggested that peptide chelated with selenium enhanced its thermal stability, this result was similar to that of corn peptide-ferrous chelate (Qu et al., 2022).

### 3.9 *In vitro* antioxidant activity analysis

**Fig. 4:**
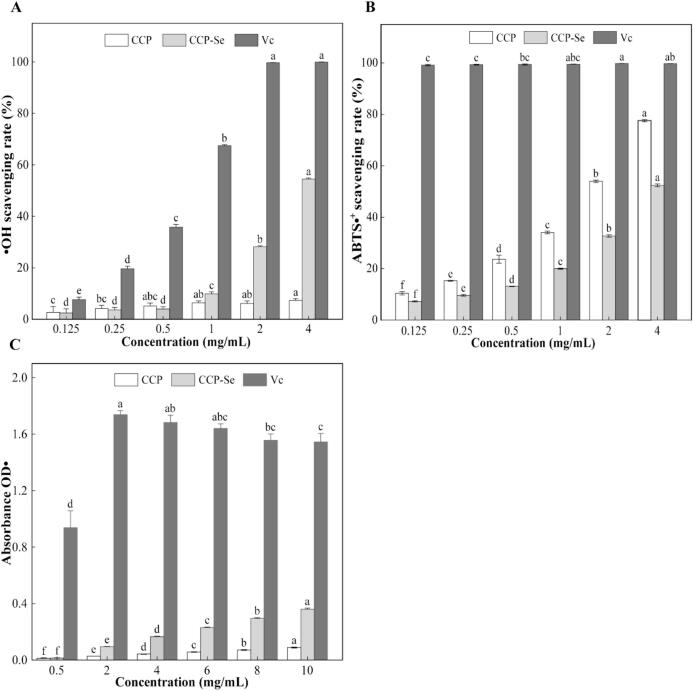
Free radical scavenging activity of CCP and CCP-Se. (A) •OH scavenging activity; (B) ABTS•^+^ scavenging activity; (C) iron ion reducing power.

Oxidative stress caused by free radical overload was considered to be the etiology of a variety of diseases, and naturally derived compounds had gained attention for their role in inhibiting oxidative stress responses (Wei et al., 2024). Antioxidants can counteract the deleterious effects of actives on cell membranes by three main mechanisms: hydrogen atom transfer (HAT), single electron-proton transfer (SET) and sequential proton-loss electron-transfer (SPLET), and the ability to chelate transition metals (Mohammadi et al., 2024). Ferric reducing ability and ABTS•^+^ scavenging ability are primarily related to SET, •OH scavenging ability is associated with the HAT mechanism of action. The •OH, ABTS•^+^ radical scavenging and the ferric reducing ability results of sample solutions were shown in Fig. 4, the antioxidant activity efficiency of the CCP-Se was positively correlated with the concentration. When the concentration of CCP-Se was 4 mg/mL, the ferric reducing ability was 0.166 ± 0.003 and the scavenging activity against •OH and ABTS•^+^ can approach 54.49 ± 0.32 % and 52.39 ± 0.54 %, respectively. Compared with the positive control, although the free radical scavenging performance was inferior to the Vc, CCP-Se still had a huge potential for regarding as an antioxidant. The effect of CCP on •OH scavenging activity was not significant in the concentration range measured, however, the scavenging activity of CCP- Se on •OH was significantly higher than that of CCP, which may be attributed to the enhanced transfer of hydrogen ions due to chelation with selenium, this was similar to previous studies (Lv et al., 2023; Ye et al., 2019). It was worth noting that CCP chelation with selenium enhanced its ferric reducing ability but decreased ABTS•^+^ scavenging rate. This may due to ABTS•^+^ was better suited to dissolve in water-soluble environments compared to Fe^3+^, when the peptide was chelated with selenium, the peptide folded more tightly, so it may result in ABTS•^+^ not being easily diffused to the hydrophobic antioxidant active groups inside the peptide. Therefore, CCP demonstrated a higher ABTS•^+^ free radical scavenging capacity than CCP-Se (Luo et al., 2022). Overall, CCP-Se was a potential and promising selenium supplement with some antioxidant activity.

### 3.10 The thermal and pH stability analysis

Decomposition occurred when the chelate was subjected to unfavorable external factors, and the mineral ions in the complex would be released into the free state. The peptide itself may also be partially hydrolyzed due to different temperatures or pH values (Zhang, Ding, and Li, 2021). Therefore, the stability of chelates can be evaluated by measuring the retention of selenium (Sun et al., 2021).

**Fig. 5:**
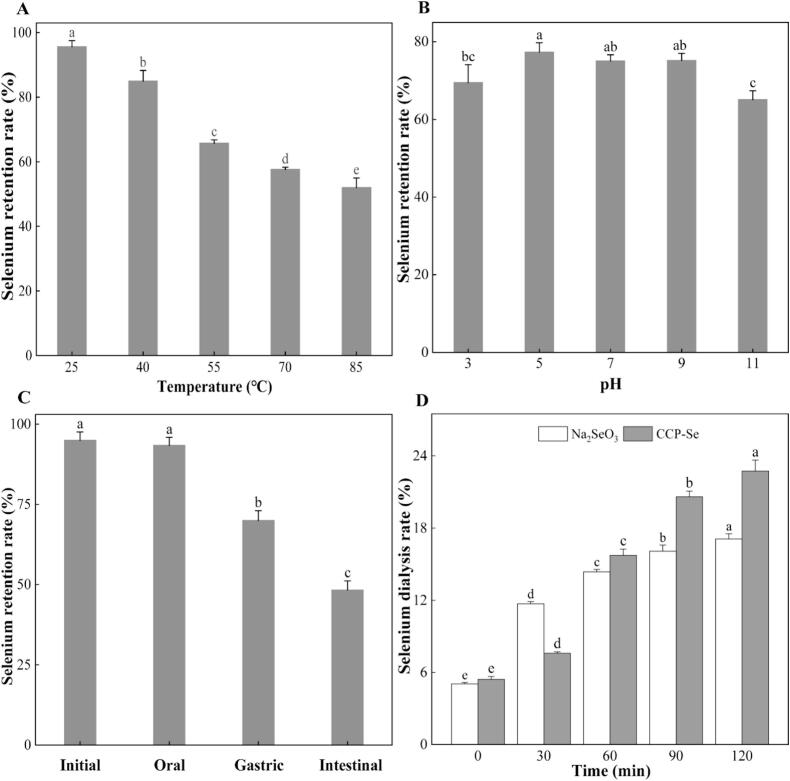
(A) The stability of CPP-Se in various temperature; (B) The stability of CPP-Se under acidic and alkaline conditions; (C) Digestion stability of CCP-Se *in vitro*. (D) Simulated intestinal permeability of CCP-Se and Na_2_SeO_3_. Different letters indicate significantly different (*p* < 0.05).

The effect of heat treatment at different temperatures on the stability of CCP-Se was evaluated as shown in Fig. 5A. The structure of the CCP-Se was relatively stable within a certain temperature range, indicated by the high selenium retention rate of CCP-Se at low temperature treatment. With the increase of temperature, the selenium retention rate of CCP-Se significantly decreased (*p* < 0.05), indicated that excessive heat treatment may destroy the spatial structure of the peptide, leading to a decrease in selenium retention rate and adversely affecting the stability of the chelate. The selenium retention rate of chelate remained above 57.64 ± 0.66 % when the treatment temperature was above 70 °C, indicating that CCP-Se had a relatively good thermal stability. When the temperature was higher than 80 °C, there was obvious precipitation in the selenium-peptide chelate, and the selenium chelation rate was significantly reduced (*p* < 0.05). This was because the structure of CCP-Se was destroyed at high temperatures, leading to the destruction of the peptide binding site and poor stability (Qu et al., 2022).

The selenium retention rate under different conditions has an important relationship with selenium uptake and utilization efficiency. The Fig. 5B showed the effect of CCP-Se on selenium retention rate at different pH conditions. When pH was in the range of 5.0–9.0, the selenium retention rate was above 75 %, and remained stable. The selenium retention rate was significantly decreased (*p* < 0.05) when the pH was reduced to 3.0 or increased to 11.0. It was speculated that H^+^ competed with selenium ions under acidic conditions, and the interaction between selenium ion and amino or carboxyl groups were weakened under alkaline conditions, which resulted in the binding amount of selenium ions reduced (Zhang, Ding, and Li, 2021). Strong acidic and alkaline conditions could lead to the conversion of selenium in the chelated state to free selenium, thus reducing the stability of peptides‑selenium chelate. Therefore, when CCP-Se was added to foods as a selenium fortifier, it should be avoided to be treated with over-acid or over-base conditions. The pH value of the human intestinal environment was about 7.2, the body temperature was about 37 °C, at which the selenium in CCP-Se was in a high stable state, so it can be beneficial to its well absorbed by the body.

### 3.11 *In vitro* simulation of oral gastrointestinal digestion stability analysis

The dietary nutrients that ingested by the body need to be digested by the stomach first, and then digested and absorbed by the intestine. Therefore, the stability during the digestive process is important to the bioavailability (Eckert et al., 2014). The selenium retention rate of CCP-Se in different digestion stages was shown in Fig. 5C, the selenium retention rate after oral, gastric and intestinal digestion were 93.35 ± 2.51 %, 69.98 ± 3.05 % and 48.24 ± 2.91 %, respectively. There was no significant change in selenium retention rate in the oral digestion phase in comparison with the undigested stage. After digestion in the simulated gastric environment, the selenium retention rate was significantly reduced (*p* < 0.05). This may be due to the high concentration of H^+^ in the stomach environment, which may compete with selenium ions for active binding sites and resulted in a lower selenium retention rate, this was in consistence with the aforementioned result of pH stability. A previous study by Zhang, Zhao, et al. (2021) reported that the gastric environment was the most important major site for the release of metal ions. Peptides undergo peptide bond breakage in strong acidic environments and further hydrolysis by pepsin, which may lead to changes in the spatial structure of the complexes, thus promoting the release of selenium ions and leading to a decrease in the stability of the complexes (Zhang, Ding, and Li, 2021). In the simulated intestinal digestion stage, the selenium retention rate decreased significantly, the reason may be that the presence of trypsin caused the degradation of the peptide, which affected its ability to bind with selenium ions (Udechukwu et al., 2018). The retention rate of selenium could be maintained above 45 % after digestion for 4 h, which indicated that CCP-Se had good digestion stability, the result was similar to sheep bone collagen peptide chelation with calcium ions (Wang et al., 2020). This suggested that CCP-Se has potential as a new selenium supplement to improve selenium absorption in the human gastrointestinal tract.

As shown in Fig. 5D, the selenium dialysis rate of CCP-Se and Na_2_SeO_3_ in the simulated intestinal digestion process increased gradually with the increase of intestinal digestion time. When the digestion time was 0–30 min, the selenium dialysis rate of sodium selenite was higher than that of peptides‑selenium chelate. When the digestion time was 30–120 min, the selenium dialysis rate of CCP-Se increased from 7.75 ± 0.12 % to 22.74 ± 0.91 %. The selenium permeability rate of Na_2_SeO_3_ only increased from 11.71 ± 0.17 % to 17.11 ± 0.42 %. The reason for this difference may be that although the selenium content of inorganic selenium was high, it was unstable when entered the acid-base environment of the intestine, whereas the chelated compound plays an important role in improving the bioavailability of selenium ions in the physiological environment of human gastrointestinal tract. On the one hand, in the pH range of the gastrointestinal tract, the peptides- selenium chelate has a certain buffering effect, which can partially resist the intestinal alkaline conditions (Li et al., 2019). On the other hand, peptides‑selenium chelate can be further decomposed into small molecules under the action of pepsin or trypsin, and re-enter the intestine for absorption, thereby the absorption rate and bioavailability of selenium were improved (Wang et al., 2015). In conclusion, CCP-Se had a significantly higher selenium dialysis rate in simulated intestinal tract than inorganic selenium salts, which was an excellent new potential selenium supplement.

## 4 Conclusion

In this study, cattle bone collagen peptides and sodium selenite were chelated to prepare CCP-Se, and the chelating capacity of selenium was 33.65 ± 0.13 mg/g. The structural analysis of CCP-Se showed that the addition of selenium led to the structural difference between CCP and peptides‑selenium chelate, and formed a compact structure after chelating with selenium. The amino nitrogen atom, carboxyl group and hydroxyl oxygen atom in the peptide chain were the important binding sites of selenium to CCP. CCP-Se exhibited excellent scavenging ability on •OH and ABTS•^+^ free radicals, which indicated its potential to be used as an antioxidant substance. In addition, CCP-Se showed a good acid-alkali resistance, heat resistance, and *in vitro* digestive stability. CCP-Se exhibited better thermal stability than CCP and better intestinal selenium permeability than sodium selenite, which may improve the bioavailability of selenium. This study provides theoretical basis for the high-value application of cattle bone and implies the potential of CCP-Se as a new effective selenium supplement. However, the specific structure, interaction and physiological mechanism of peptides‑selenium chelate *in vivo* are required to be further studied for the application of peptides‑selenium chelate in foods and medicines.

## CRediT authorship contribution statement

**Jian-Ming Li:** Writing – review & editing, Supervision. **Wen-Jun Wang:** Writing – review & editing, Supervision, Funding acquisition. **Hui Chen:** Formal analysis, Data curation. **Su-Yun Lin:** Formal analysis, Data curation. **Xin-Yi Mao:** Methodology, Formal analysis. **Jun-Min Yu:** Methodology, Formal analysis. **Ling-Li Chen:** Writing – review & editing, Supervision, Methodology, Funding acquisition.

## Declaration of competing interest

The authors declare that they have no competing financial interests or personal relationships that could have appeared to influence the work reported in this paper.

## Data Availability

Data will be made available on request.

## Appendix A Supplementary data

Supplementary material Image 1
