# Supplementary material for: Characterization, in vitro antioxidant activity and stability of cattle bone collagen peptides‑selenium chelate
Source: Food Chem X. 2024 Aug 30;23:101789. doi: 10.1016/j.fochx.2024.101789 (PMC11639323; doi:10.1016/j.fochx.2024.101789)
Supplement: Supplementary file 1 — Supplementary material [file mmc1.docx]

**Supplementary material: The optimization of preparation conditions of CCP-Se**

Fig. S1 The main factors affecting the selenium binding capacity, (A) pH; (B) Temperature; (C)Time; (D) The mass ratio of CCP/Se. Different letters indicate significantly different (*p* < 0.05).

Fig. S2 Response surface plots and contour plots (A, B and C) showing the effect of pH (X1), Temperature (X2), mass ratio of CCP/Se (X3) on selenium binding capacity.

**Fig. S1**


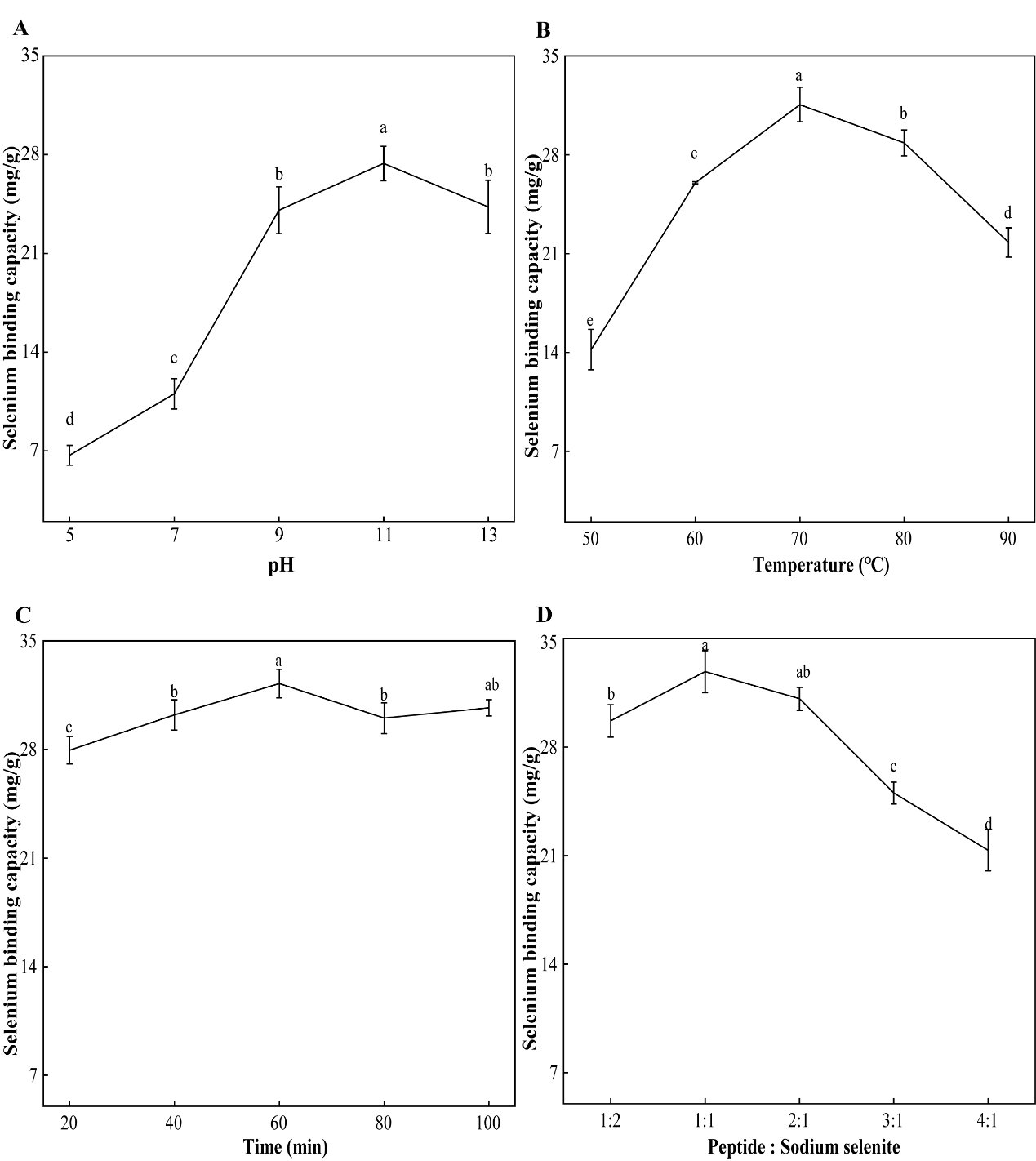


**Fig. S2**


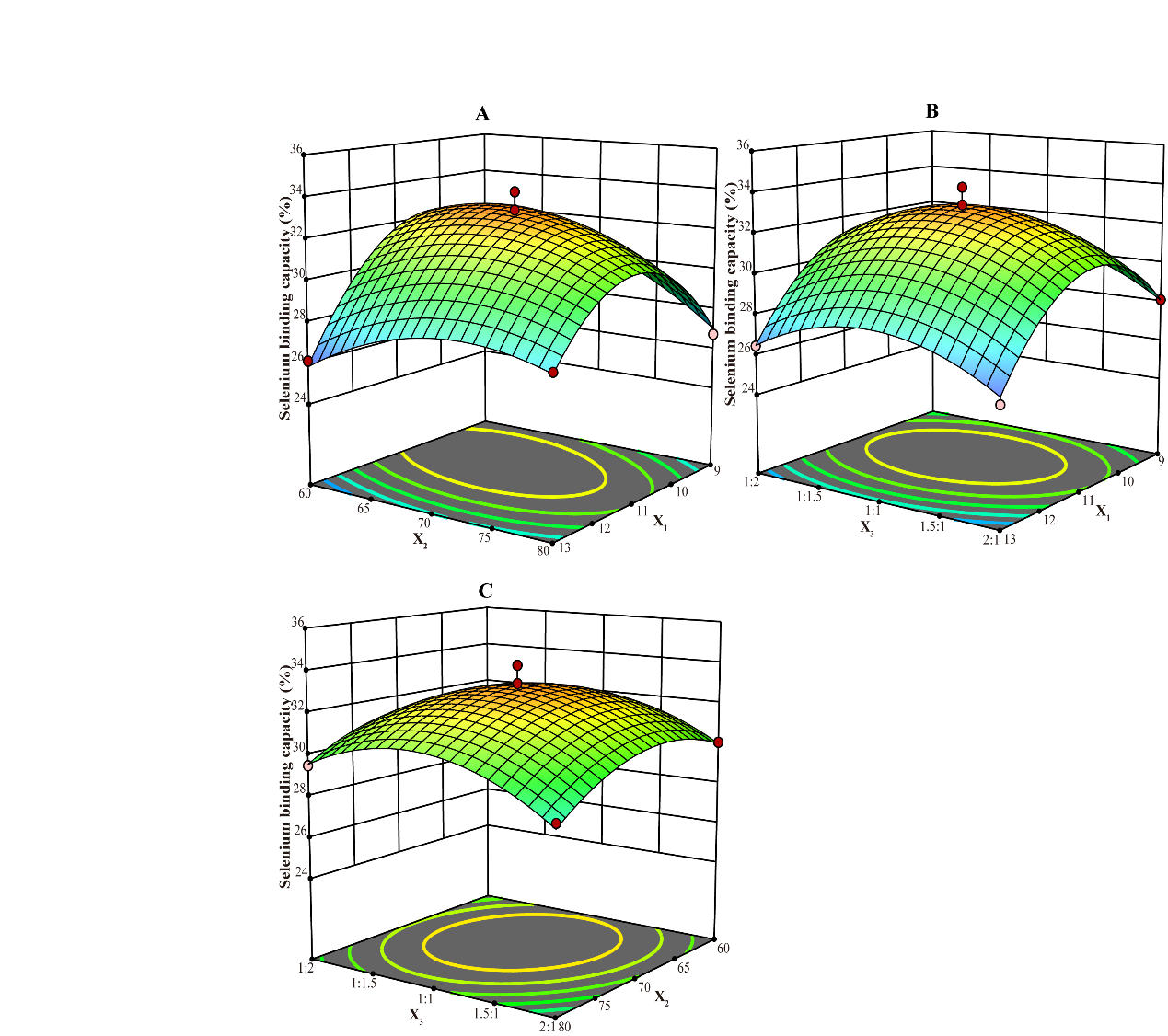


**Table S1**

Coded values and corresponding actual values of the variables used in Box-Behnken design.

| Value |  | Level |  |
| --- | --- | --- | --- |
|  | -1 | 0 | 1 |
| X_1_ | 9 | 11 | 13 |
| X_2_ | 60 | 70 | 80 |
| X_3_ | 1:2 | 1:1 | 2:1 |

X_1_: pH, X_2_: Temperature, X_3_: mass ratio of CCP/Se.

**Table S2**

Analysis of variance (ANOVA) for experimental results.

| Source | Sum of Squares | DF | Mean Square | *F*-value | *P*-value | *Inference* |
| --- | --- | --- | --- | --- | --- | --- |
| Model  X_1_  X_2_  X_3_  X_1_X_2_  X_1_X_3_  X_2_X_3_  X_1_^2^  X_2_^2^  X_3_^2^  Residual  Lack of Fit  Pure Error  Core Total  R^2^=0.9717 | 106.81  10.62  2.54  0.9605  7.65  0.0802  0.3053  53.16  9.30  14.81  3.11  0.5667  2.54  109.92  AdjR^2^=0.9353 | 9  1  1  1  1  1  1  1  1  1  7  3  4  16 | 11.87  10.62  2.54  0.9605  7.65  0.0802  0.3053  53.16  9.30  14.81  0.4445  0.1889  0.6362 | 26.70  23.89  5.72  2.16  17.20  0.1805  0.6869  119.60  20.91  33.31  0.2969 | 0.0001  0.0018  0.0480  0.1850  0.0043  0.6837  0.4346  < 0.0001  0.0026  0.0007  0.8269 | Significant  Not significant |
